# Microfluidics as a Ray of Hope for Microplastic Pollution

**DOI:** 10.3390/bios13030332

**Published:** 2023-02-28

**Authors:** Emre Ece, Nedim Hacıosmanoğlu, Fatih Inci

**Affiliations:** 1 UNAM-National Nanotechnology Research Center, Bilkent University, Ankara 06800, Turkey; 2 Institute of Materials Science and Nanotechnology, Bilkent University, Ankara 06800, Turkey

**Keywords:** microplastic pollution, microfluidics, separation, toxicity

## Abstract

Microplastic (MP) pollution is rising at an alarming rate, imposing overwhelming problems for the ecosystem. The impact of MPs on life and environmental cycles has already reached a point of no return; yet global awareness of this issue and regulations regarding MP exposure could change this situation in favor of human health. Detection and separation methods for different MPs need to be deployed to achieve the goal of reversing the effect of MPs. Microfluidics is a well-established technology that enables to manipulate samples in microliter volumes in an unprecedented manner. Owing to its low cost, ease of operation, and high efficiency, microfluidics holds immense potential to tackle unmet challenges in MP. In this review, conventional MP detection and separation technologies are comprehensively reviewed, along with state-of-the-art examples of microfluidic platforms. In addition, we herein denote an insight into future directions for microfluidics and how this technology would provide a more efficient solution to potentially eradicate MP pollution.

## 1. Introduction

Throughout history, humanity has interacted with its surroundings to produce goods and create a sustainable living system to maintain its population at the best possible level. This search for a sustainable life has required the utilization of many environmental resources, including rocks, clay, wood, metals, and many other organic or inorganic materials to use as blocks to support human activities [1]. With the increasing size of the population, alternative materials have been incorporated into production schemes to fabricate goods more efficiently and in a shorter period of time. “Plastic” is an ultimate polymer material discovered to answer the need to produce goods in the shortest possible time and in the largest quantities possible [2]. Briefly, the main structure of plastic consists of polyethylene (PE), polystyrene (PS), polyamide (PA), nylon, and many other chemicals. The polymers can be classified as thermosets and thermoplastics, depending on the bond structure formed by the long-chain carbon constituents. However, although plastic became a major source of commodity production in the 1950s, and has become a dominant source of production since the 2000s, it presents an overwhelming problem—MP pollution—that is now affecting every living organism on the planet [3,4,5]. The main reason for this global catastrophe is the uncontrolled release of plastics into nature. The methods for the production of many everyday tools and products, such as food packaging, textiles, fishing equipment, and various types of agricultural equipment, as well as face masks and medical supplies, incorporate large volumes of plastics that are either produced as MPs or become MPs as a result of environmental effects. Considering the yearly production of plastics (350 million tons in 2017) and an estimated release to nature of 60%, MP pollution is considered one of the most significant environmental issues to have been faced by humanity, and that will be faced in decades to come [6]. The scope of the MP problem is not only limited to plastic remnants around the globe; it also extends to being a factor affecting all known environmental cycles. Changes in the metabolism of organisms, affecting feeding types, and reducing viable resources that are necessary for wild animals are also serious outcomes of the MP problem. MP pollution has become a primary environmental concern following the realization that plastic waste is greatly affecting the marine environment. Back in the 1970s and 1980s, for instance, scientists warned global authorities against the production of marine plastic pollution as a significant environmental issue [6]. However, since the 1990s, and with the beginning of the new millennium, plastic waste floating in the marine ecosystem has become an immense floating mass, with a size that is greater than many countries’ territorial sizes [7]. In recent years, it has also been realized that sheared plastics or micron-sized plastics do not dissolve quickly in nature; in fact, MPs are passing through the food and water cycles from plastic waste to animals and from animals to humans [8].

An increasing body of evidence confirms that MP intake into humans is possible via many routes, including skin contact, food digestion, and even breathing. Starting from the marine ecosystem and food sources produced using MP-contaminated water, the food chain is highly exposed to MP, which may have significant negative effects on it [3,9]. In addition, seafood includes raw or processed fish, shell-bearing organisms, and even marine plants. Many organisms are exposed to MPs floating on the sea surface, and due to their respiratory system, which depends on soluble oxygen, it is necessary for them to pass seawater through their respiratory organs. This passage of water causes seafood to be potentially at risk of MP contamination; in fact, many seafood sources carry dangerous levels of MPs [10]. Considering the challenges in detecting nanoplastics, the effects of seafood contamination will be notably high in persons that primarily consume seafood. Many other food sources possess great potential to be reservoirs of micro- and nanoplastics that could threaten human health [6].

This review aims to elucidate the separation and characterization of MP pollution via microfluidic platforms by expressing the jeopardous situation of MP pollution around the world. Unlike the reviews in the literature on the analysis of MPs using microfluidic platforms, which typically rely on only a single source, in this study, MP analyses are compiled from many sources, including soil, water environments, and food. In addition to gathering information from a number of distinct sources, we present the established strategies for the detection, isolation and removal of MPs using state-of-the-art microfluidic platforms and integrating technologies, which provide robust solutions aimed at surmounting the issues caused by MP pollution. By also investigating types of plastics and the future directions on MP pollution, we herein aim to create an awareness of this increasingly serious issue among the scientific community and society.

## 2. Classification of Microplastics

MPs are defined as any plastic structures with a diameter smaller than 5 mm, although their size can be as tiny as nano-sized objects [11]. In addition, MPs can be found that possess distinct shapes (e.g., beads, fibers), or belonging to any other type of regular or irregular structure, including fragments, flakes, films, lines, fibers, pellets sponges, and foams [11,12]. To understand the precarious status of MPs and their types, along with their properties, MPs have been divided into two major classes: (i) primary and (ii) secondary MPs [13]. The most frequently encountered members of the primary class include the microbeads contained in personal care products, and which are widely found in plastic pellets, fibers, dish sponges, and even the rubbers used in running tracks at schools, and they generally contain PE, polypropylene (PP), and PS polymers. However, these MPs have been found to be less common than secondary MPs in marine systems. They are frequently used due to their chemical resistance, affordability, and mechanical strength qualities [11]. Specifically, these toxic chemicals are able to more easily enter the skin due to the absorption capabilities of microbeads [14]. Secondary MPs are formed by the breaking down of plastic products produced in large volumes into smaller pieces as a result of external factors [15]. Even if physical shearing and UV radiation are dominant factors for plastic shearing, these are the forces driving the generation of MPs that are not caused by human activities [15]. Plastic bags, bottles, fishing equipment, disposable products, and tea bags are also among the most common sources of secondary MPs [16]. PE, PP, and polyvinyl chloride (PVC) polymers are commonly found among MPs that have been broken down into smaller pieces by environmental factors, including UV rays and ocean waves, and which are the main cause of MP toxicity via seafood and water origin [5,15].

## 3. Conventional Microplastic Detection Methods

MP pollution has revealed the necessity for adapting technological tools and methods to battle this global problem. Technological developments critically depend on the types and features of MPs, and the perseverance of the detection processes [17]. New modalities and technologies have been introduced over the course of decades to reduce the cost and time required, while increasing the yield of sampling, separation, and detection of MPs [18,19,20,21]. In conventional approaches for MP analysis, a two-step protocol was applied, which included (i) standardized sampling and preparation of the samples and (ii) quantitative analysis of the MPs.

Sample collection (i.e., via sieving [8], pumps [22], neuston and plankton nets [23,24], or forceps [25]) is the first step in identifying MPs inside complex samples. The surplus components, such as organic matter, which affect MP concentration and the number of particle tests, should then be removed using extraction and separation techniques to prevent false positives and increase specificity [26]. Extraction and separation techniques include digestion procedures (such as acid [9], alkali [27], oxidizing reagent [28], and enzymatic digestion processes [29]), filtering techniques [30], solid phase microextraction (SPME) [31], magnetic extraction [32], microwave employment [33], ultrasonic methods [34], gas chromatography [35], and gel permeation chromatography [36], which provide efficient separation and clarification of MPs from the sample they are contaminating.

**Figure 1:**
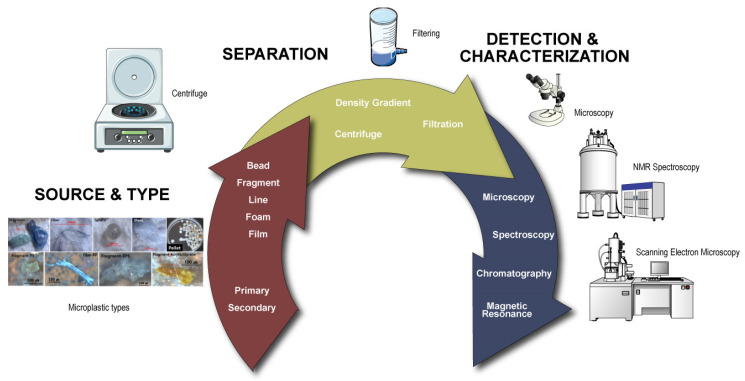
A schematic diagram of the microplastic analysis process, from source and type to analysis techniques. NMR spectroscopy, scanning electron microscopy (SEM), and stereo microscope icons by DBCLS (https://togotv.dbcls.jp/en/pics.html) (accessed on 3 February 2023) were licensed under CC-BY 4.0 Unported https://creativecommons.org/licenses/by/4.0/. Filtration-1 and centrifuge icons by Servier (https://smart.servier.com/) were licensed under CC-BY 3.0 Unported https://creativecommons.org/licenses/by/3.0/. Microplastic types was reprinted with permission from [46]. Copyright 2018, Elsevier.

Next, the extracted MPs are detected and classified using analytical techniques such as spectroscopic, optical, or chemical approaches (Figure 1). The types of equipment employed most frequently for MPs include gas chromatography (GC) [37], mass spectrometry (MS) [38], X-ray photoelectron spectroscopy (XPS) [39], Fourier-transform infrared spectroscopy (FT-IR) [40], and Raman spectroscopy (RS) [41]. Several extraction procedures and gravimetric techniques can also be combined to improve the sensitivity and selectivity of detection. Chemical-based techniques, including RS and FT-IR, are utilized for a myriad of MPs, such as nylon (NY), polyethylene terephthalate (PET), PE, PP, and PVC, which are frequently observed in marine products and food [1]. FT-IR is a crucial technique, since it can demonstrate structural fingerprints and detect light refractions from functional groups of molecules [42]. The measurements become more complicated when the size of the pollutants is considerably larger than what can be quantified by the instrument. In such cases, pyrolysis (pyr) is used to deal with the issue by shattering the polymers into tiny pieces. The pyr-GC/MS technique increases the detection yield of MPs, and has the capacity to rapidly separate and isolate MPs [19]. On the other hand, instruments involving optical [43], fluorescence [44], and electron microscopy [45] are crucial visualization methods for MPs.

Despite their advantages, the aforementioned approaches also have some drawbacks that include high cost, the need for qualified personnel, and the limitations of the field applications. These shortcomings have paved the way for the development of more effective techniques in a wide range of fields. In order to obtain a well-developed scheme for field-deployable, low-cost, and easy-to-operate MP detection and separation systems, alternative technologies need to be developed. Due to advantages that include affordability, portability, flexibility with respect to sample volume, integrability with many spectroscopic and microscopic methods, rapid turnout, and high precision, microfluidics is becoming a prominent technology in the separation and characterization of such contaminating and detrimental MPs [47,48,49]. In addition to these techniques, MP detection and characterization strategies span a wide range of tools in the literature. In the table below, common approaches to MP characterization strategies reported in the literature are listed, along with their advantages, disadvantages and the efforts required for them to be able to work with different sample types (Table 1). Although the traditional methods present intrinsic challenges and disadvantages, the combination of these methods with microfluidic systems is just as inevitable. In addition to the use of microfluidic systems and conventional methods together, technologies that can be directly integrated into microfluidic systems could lead to both the portability of microfluidic systems and the possibility of the in-field application of very well-known procedures. For example, instant detection assessments can be performed using miniaturized detection systems by integrating them into a microfluidic system [50], and in addition, these analysis methods can be expanded to larger volumes with techniques such as capillary electrophoresis, capillary electrochromatography, and high-performance liquid chromatography, which are included in the input/output part of the microfluidic system or integrated with their microfluidic channels [51]. This improvement in both detection and separation processes would help improve the accuracy of analysis results, especially in large sources such as water. Moreover, by miniaturizing laser optic microscopes, the integration of membranes or nanomembranes developed at smaller scales into these microchannels could motivate the use of technologies and methods such as microscopy and chromatography within microfluidic systems. Instant results can also be obtained with microfluidic platforms integrated into the interior of large-scale devices such as those used for FT-IR [52]. As a result, microfluidic systems and conventional methods are mutually complementary, allowing results to be obtained with higher efficiency and shorter assay times in the process of MP detection and separation.

## 4. Microfluidic Platforms to Tackle MP Pollution

**Figure 2:**
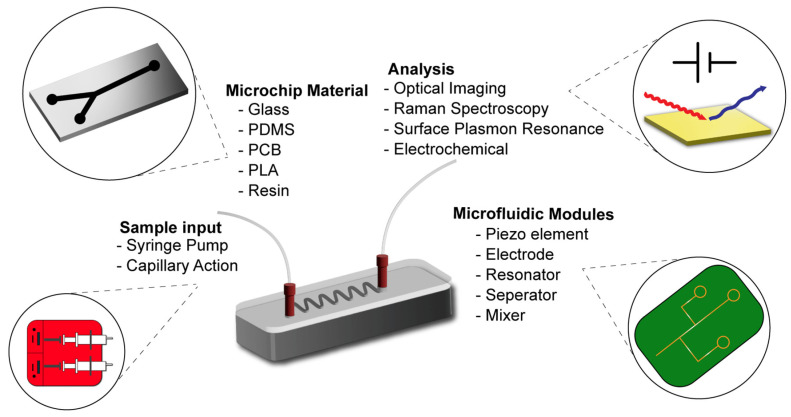
The versatility and modularity of microfluidic strategies for MP research.

Microfluidics aims to design and utilize micrometer-sized flow systems that have applications in nearly every field of scientific research. This technology can be utilized in MP detection and separation strategies in many different ways with the support of different experimental strategies (Figure 2). As their essential property, microfluidic systems allow researchers to manage flow rates and integrate a vast array of manipulation strategies (mixing, separation, droplet formation, etc.) [61,62,63]. This property is very well suited for MP studies, since the residency time of MPs is generally different from that of other particles in environmental samples [64]. This separation process is made possible by the manipulation of the fluids, which is a fundamental aspect of microfluidic systems. For example, in the case of the Dean Flow effect in curved microfluidic systems, particles are separated on the basis of their sizes [10]. In particular, in such systems, an analysis of the MP separation occurring in this flow can be performed with the help of instantly reduced sensors. As highlighted in the previous section, the integration of such sensors can enable better observation of this pre-existing advantage of the flow, resulting in the higher-accuracy detection of separated particles. Considering these advantages, there have been a number of different studies in the literature that have incorporated the operating principles of microfluidics, utilizing the power of these characterization and separation strategies to design a new field of study into microfluidics focusing on MP.

## 5. Microfluidics as a Separation/Recovery Tool for MPs

**Figure 3:**
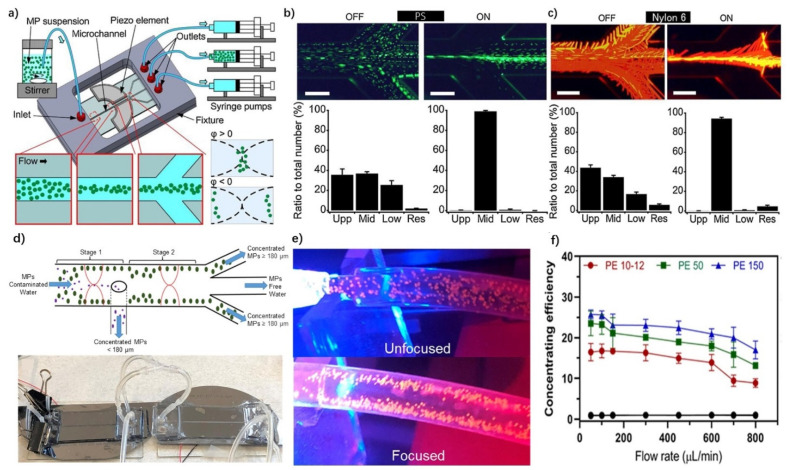
Physical separation methods utilized for the separation and collection of MPs. (**a**) Illustration of the acoustic focusing integrated microfluidic platform, demonstrating the mobility of MPs with the application of the focusing. (**b**,**c**) The collection yield of PS and Nylon 6 MP was higher when applying acoustic focusing. Reprinted with permission from [65]. Copyright 2020, Elsevier. (**d**) Removal capacity for MPs larger than 180 µm, as well as an image of the microfluidic system. (**e**) Direction of MPs using acoustic focusing. (**f**) Analysis of the relation between the concentration efficiency of PE and the flow rate. Reprinted with permission [66]. Copyright 2022, Elsevier.

MP research and identification strategies often start with the separation of MPs from complex media in order to increase the efficiency of characterization and identification. Studies in the literature focusing on isolation and separation have recently emerged that incorporate different physical and chemical strategies. With these studies, the identification and characterization process of MPs are facilitated through the separation of these particles from each other and their collection in a certain location. Among these studies, Akiyama et al. isolated PS, nylon 6, and PET as MPs in the middle of the channel of a Pyrex glass microfluidic system [65]. In that study, a set of piezoelectric actuators was used to create acoustophoretic force by exerting bulk acoustic force with the generated vibrational waves on the microfluidic device walls (Figure 3a–c). With this kind of exerted force, the particles within the liquid could be collected at a specific point, known as the “zero-displacement point”. For MPs with a diameter of about 5 µm, this collection method was quite effective; however, it was found that certain fibers could not be collected because they attached to the sides of channels. The MPs in the wastewater were efficiently and quickly collected in this study [65]. MPs of various sizes could be collected, and the proportion of the collected MPs could be increased by altering parameters such as frequency and flow rate. Considering the efficiency of this technique and the fact that it was possible to collect microplastics and microfibers as small as 5 µm, this technique could be considered for the design of large-scale purification systems for microplastic separation.

Along a similar line, PS, PE, and polymethyl methacrylate (PMMA) MPs varying in size between 6 and 300 µm in diameter were isolated using a microfluidic system containing steel tube channels that were 484 µm in width [66]. In this configuration, MPs with diameters smaller than 180 µm could be precisely isolated (Figure 3d–f). The effect of medium density on this isolation was investigated. Flow rates between 50 µL/min and 800 µL/min were investigated, and MPs were detected in higher concentrations at lower flow rates. At a flow rate of 800 µL/min, about a 50% reduction in the concentration efficiency of the MPs was observed. On the other hand, real-time monitoring using a microscope is not possible due to the lack of light transmittance.

In another approach, the charge properties of the MPs can be utilized for the separation process instead of using acoustic forces. Thompson et al. used the electrical properties of microplastics, which are generally charged, and a glass/PDMS microfluidic chip incorporating previously implemented bipolar electrodes produced with photolithography [67]. By creating electric field gradients inside the microfluidic channel, this system is able to separate charged MPs without any need for labeling or special buffering. A separation efficiency greater than 99% was obtained.

For non-charged MPs, the mass-based separation method, which is commonly used in microfluidic systems as a result of the Dean flow effect, was employed by Chen et al., 2022 [10]. A spiral microfluidic system was made with a PDMS channel designed to separate MPs in a size-dependent manner based on the inertial lift force and the Dean drag force created by the flow inside the microfluidic channel. Considering the ease of production of the spiral channel and the reported efficiency, this technique could also be applied in high-throughput MP-capturing studies in device-limited environments. This overall strategy is also capable of recovering 90% of the microplastics introduced with the various sample types with a minimal volume requirement.

In addition to mass-based separation methods, the physical properties of these MPs, such as size and shape, are other advantages of capturing them via nanofibril membranes. For instance, Leppänen et al. designed a plant-based cellulose nanofibril (CNF) system that was capable of capturing microplastics and nanoplastics from aqueous solutions [68].The hygroscopic properties of nanocellulose networks in microfluidic systems facilitated the capillary and diffusion properties of the water. The micro- and nanoplastics remained in the network, while the water diffused through it. Anionic and cationic PS (1.0 µm and 100 nm) and PE (38–45 µm) micro- and nanoplastics were therefore trapped and characterized via fluorescence microsopy, scanning electron microscopy, and quartz crystal microbalance with dissipation (QCM–D) at various pH levels and under various ionic conditions of the CNF, TEMPO–CNF, and regenerated cellulose (RC) networks. While the capture yield of the PS particles remained constant throughout a range of pH values, it varied depending on the ionic conditions (40 mM and 200 mM NaCl). For both CNF and TEMPO–CNF, the adsorption of nanoplastic particles increased with increasing values of ionic strength. As a consequence, this study provided insight into the significance of the cellulose network structure and its circumstances on the capture of plastics from natural resources, inspiring in-field applications for the collection of micro- and nanoplastics [68].

Separation procedures for MPs have been improved by utilizing additional techniques such as particle trapping. Using a microscope that was able to detect particles at high speeds, Kitagawa et al. created a microfluidic platform to isolate and analyze PE MPs with diameters between 0.96 and 36 µm [69]. Pillars of four different sizes and shapes (circular, square, diamond, and triangular) were used in this study to compare the impact of shape and size on MP capture. The square-shaped pillar exhibited an efficiency of 100%, which was better than that demonstrated by the other pillars, all of which demonstrated efficiency values >70%, and this efficacy could be enhanced by making the pillars larger. Additionally, at a flow rate of 600 μL/min, the hydrophilicity of the pillar medium was investigated under both hydrophilic and hydrophobic conditions. As a consequence, a square-shaped pillar with larger dimensions and hydrophobic surroundings demonstrated the highest MP catching capacity. The application range of the MP separation and detection methods using microfluidic systems can be expanded through the integration of these types of strategies.

The capture and separation of MPs have benefited from basic features of microfluidic systems such as liquid manipulation, and from the addition of necessary features into the channels. In addition, separation can be performed using external forces such as acoustic force and electric fields, and owing to these separations, the processes of characterization and identification can be facilitated.

## 6. Detection Strategies for MPs Integrating Microfluidics

The preceding section emphasized the significance of separating MPs from the resources in which they are found, and from each other. This section describes, in addition to these separation methods, separation strategies and how all of these separated particles can be analyzed. Many approaches, including the use of fluorescence properties, optical analysis, and chemical characterization, have been employed in these analysis processes, along with the assistance of appropriate conventional methods.

One of the most frequently employed methods for MP detection is the use of a hydrophobic dye called Nile Red, which can be easily detected due to its fluorescence when attached to MPs. In a recent study, Mesquita et al. produced a low-cost PE and PS detection device with the advantages of high efficiency and continuous imaging through the addition of Nile Red solution to a 3D-printed PDMS microfluidic system [64]. In that study, the flow rate and temperature increased from 3.26 µL/min to 7.82 µL/min and from 25 °C to 80 °C, respectively. The highest fluorescence intensity was obtained at the lowest flow rate (3.26 µL/min) and at the highest temperature value (80 °C). As a result, non-spherical MPs demonstrated better fluorescence activity. In a similar study, PS, PP, and PE MPs were detected using Nile Red solution in a PMMA-based microfluidic system [70]. FT-IR analysis showed that the match rates of the MP particles with their spectra were 0.79, 0.91, and 0.95. However, the analysis results were affected by the attraction of microplastics to each other, whereby a portion of the IR waves was not able to reach the other side of the grouped particles. 

**Figure 4:**
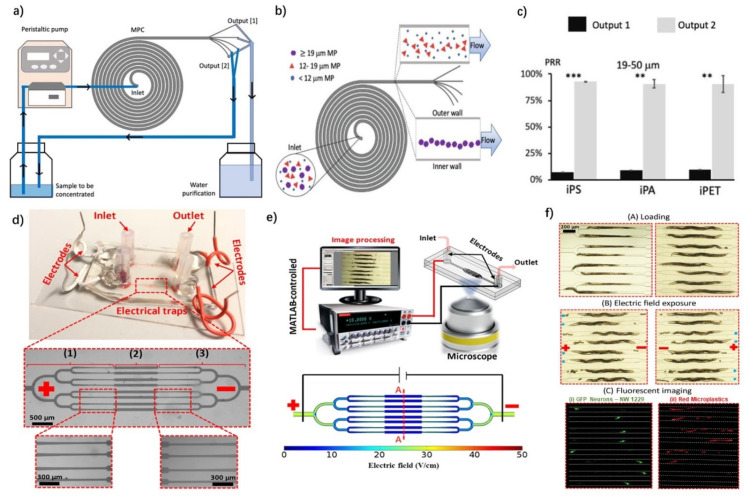
Illustration of the acoustic focusing separation method and a demonstration of its results. (**a**) A feedback loop system is integrated into the microfluidic system for continuous concentration and purification of MPs. (**b**) MPs are separated and collected according to their size differences. (**c**) High-yield separation and collection of PS, PA, and PET with diameters between 19 and 50 µm was achieved. Reprinted with permission from [10]. Copyright 2022, Elsevier. (**d**) A microfluidic system was designed with 8 channels for the investigation of the effect of MPs on the egg-laying of worms. (**e**) The system incorporated an inverted microscope for real-time monitoring. (**f**) The experimental process was demonstrated, and fluorescence imaging (green: GFP neurons and NW1229 worms; red: worms that had ingested MPs) was utilized. Reprinted with permission from [76]. Copyright 2021, Elsevier.

Recent efforts to develop MP detection strategies also include sophisticated devices for the detection and characterization of MPs that also incorporate the advantages of microfluidics. In the study by Takahashi et al., 2021, the authors measured coherent anti-Stokes Raman Scattering (CARS) and included two-photon excited autofluorescence (TPEAF) signals for the detection of separated microplastics and biological particles [71]. Here, a quartz glass flow cell was used to reduce the background noise during the imaging process, and the differential signal between the CARS and TPEAF signals was used to generate 2D images of the MPs and algae as a unique strategy for the continuous monitoring of MPs in a specific fashion. The results of this study demonstrated that microfluidic-assisted CARS signals could be used to successfully identify PMMA, PS, and Low-Density PE microplastics with a variety of sizes ranging up to the millimeter scale. Additionally, the combination of TPEAF with CARS also made it possible to distinguish algae cells from microplastics, since only algae emit TPEAF signals. On the other hand, in the study by Colson and Michel et al., 2021, impedance spectroscopy was also employed to selectively detect MPs and biological particles of small size by tracking the changes in impedance from a microfluidic channel [72]. Unlike previous efforts, this study utilized custom-made PCBs and an impedance measurement circuit to build a microfluidic system. By using polyethylene beads with a range of sizes (212−250 μm, 300−355 μm, 425−500 μm, 500−600 μm, 600−710 μm, and 850−1000 μm), the system was able to recover 90% of the beads used, distinguishing microplastics from biological material with an error rate as low as 1%. In a similar effort to establish a continuous MP monitoring system, Pollard et al. 2020 demonstrated the use of a resistive pulse sensor (RPS) with a 3D-printed microfluidic system embedded with a silver electrode [73]. In this study, the authors not only established a durable and reusable sensor system, they also achieved a detection limit as low as 2 μm and distinguished MPs from algae via the effect of porosity on the measured impedance. A PDMS-based microfluidic system with a 400 µm channel width was developed by Wu et al. for the determination of PE MPs in soil, and these impurities were captured with a film thickness that was dependent on the number of capillaries [74]. In this study, in addition to this determination method, which increased in effectiveness with increasing film thickness, the motion of MPs was investigated at various flow velocities, including 42 µm/s, 428 µm/s, 960 µm/s, and 2096 µm/s, and particle volume fraction. The higher flow rates and greater film thicknesses resulted in the capture of more PE MP on the film. In a related study, a microfluidic platform was effectively used with the integration of sensors [73]. The size and the concentration of carboxylated PS MPs originating from the use of tea bags were characterized using a microfluidic platform integrating RPS. The results showed that the MPs in the tea bags could be detected, with an average size of 21.9 µm and a concentration of 6.52 × 10^4^ particles/mL. To enhance the visualization of the structure and number of MPs, Cacace et al. combined a microfluidic system with a 3D hologram microscope, which the authors used to determine 0.2% wt/wt suspension of low-density polyethylene (LDPE), PS, and PP MPs [75]. This study used a microfluidic device to separate the pollutants, and integrated it with a microscope to evaluate the size and shape of the pollutants. As a result, the largest MPs in the solution were detected as possessing dimensions of 125 × 81 µm. In another study, 10 µm PMMA and 80 µm PS MP were isolated from one another based on their sizes using a passive size sorting approach in a microfluidic system with a reservoir width of 90 µm [48]. After the separation process, the types and sizes of the MPs were characterized using Raman and FT-IR spectroscopy. PMMA MPs with a size of 10 µm were observed in every reservoir, while PS MPs with a size of 80 µm were detected in only one reservoir. In another study, PS, PA, and PET MP solutions with densities ranging from 0.9 to 1.5 g/cm^3^ were isolated using a portable microfluidic system to overcome the low efficiency of methods such as density separation and filtration (Figure 4a–c) [10]. The MPs were collected using the sample collector located at the outlet of the microfluidic system and classified on the basis of their size properties, as follows: <8 µm, 10 µm, 12–19 µm, 21 µm, 25 µm, and 27–50 µm. The particle recovery rates (PRRs) demonstrated that pollutants with a size of 19–50 MP were removed effectively (>90%). Then, these MPs were characterized using optical photothermal infrared spectroscopy (O-PTIR).

Apart from these systems, advanced methodologies including terahertz metamaterials have also been incorporated in MPs–oriented microfluidic research. In another MP detection study, rectangular ring-shaped wells with a width of 3 µm and a depth of 10 µm were fabricated inside the channels of a microfluidic system [77]. PS with different solution densities (2 × 10^8^, 1 × 10^9^, 3 × 10^9^, and 6 × 10^9^ g/mL) were created and accumulated in the wells through interaction with hexamethyldisilazane (HMDS), which was coated on the surface of the wells. Then, PS densities were determined with great accuracy and at low cost using terahertz spectroscopy. The results indicated that the capture of PSs in the gap Increased the resonant frequency and caused a shift in the resonant frequency.

## 7. Microfluidics as a Toxicity Screening Platform for MPs

As a different approach from conventional separation and detection techniques, the toxic effects of MPs can also be examined using microfluidic chips. PS, for instance, might have a toxic effect on living organisms in the soil [78]. In this regard, Youssef et al. examined the effects of glucose and PS MPs at concentrations of 100 mg/L and 1000 mg/L on the egg-laying of Caenorhabditis elegans (C. elegans), a worm species, using a microfluidic system. This microfluidic chip was designed with eight channels that could be monitored by means of fluorescence imaging (Figure 4d–f) [76]. The results showed that PS MPs at a concentration of 1000 mg/L significantly reduced the egg-laying efficiency of the worms and caused reductions in body size. This consequently emphasizes the degree to which MPs interfere with living organisms in the soil. In addition to the effects of MPs on living organisms in the soil, the impact of PS MPs on thrombosis was investigated using a microfluidic system [79]. By observing PS particles using a fluorescent dye and an optically assisted thrombus platform, the impact of MPs on the vascular system was examined. A 1 mL sample of human blood was mixed with 1 µg PS, and the results were obtained at different times (5 min, 10 min, 20 min and 30 min). Decreased fibrin binding to platelets was observed, which demonstrates the detrimental impact of MPs manifested in the development of thrombus, and more serious thrombus was observed following long-term exposure. This occurred due to the bonding of MPs and fibrins, which might result in serious disorders related to MPs. Overall, the advantages and disadvantages of all of the studies mentioned above, as well as their incorporated measurement and production strategies, are elaborated in Table 2.

## 8. Conclusions and Future Perspectives

Despite recent efforts to establish advanced strategies to battle MP pollution, handling the global MP problem poses a significant challenge to humanity. In order to deal with pollutants, many technologies have been developed, and their deployment in the field has been attempted for decades. Nevertheless, there are some stumbling blocks in the current practice of MP research that require optimization and adaptation to achieve deployment in the field. On the other hand, microfluidics has been widely employed as a new approach, and has become a ray of hope for the rapid separation and detection of molecules. Remarkably improved sensitivity and adaptability for low amounts/numbers of materials or molecules make this technology a driving tool for addressing the problems presented by earlier technologies for dealing with MP pollution. Moreover, microfluidics possesses essential advantages, including in-field utility and reduced processing time, that underscore the usability of this technology. However, it cannot be claimed that all of these processes can currently be controlled by microfluidic technology. This is because these approaches have some inherent drawbacks that are similar to those of conventional technologies. Even though studies addressing these drawbacks are ongoing, the results acquired with each new step are inspiring advances, serving as a source of encouragement for future investigations. For instance, the adhesion of some MPs to the microchannel surface, the possibility of air bubbles and leakage, reducing the signal/noise ratio during miniaturization, the inability of collecting and separating large volumes of these impurities, and the significant deficiencies in standardization are the main drawbacks of this technology [80].

Due to the increasing extent of MP contamination, which may become more hazardous, in addition to having direct impacts on the body, the severity of this threat to nature and human health is increasing drastically [81]. It should also be noted that the advantages of MP–microfluidics assessment for various resources contaminated with MPs could directly impact prevention strategies or interventions related to hazardous pollutants. For observation and detection, it is crucial to isolate and collect these pollutants, which can be simultaneously present in a variety of sizes. A myriad of isolation and detection techniques have been reported in the literature, shedding light on the latest techniques [82]. Many studies in the literature introduce new materials for microfluidic production, along with their fabrication techniques. PDMS, PMMA, and glass materials, which have excellent optical transmittance, are frequently utilized for such platforms. The optical features of these materials facilitate the real-time observation of targeted molecules or processes. For that reason, visualization techniques such as microscopes and chemical characterization methods such as FT-IR can be integrated into portable devices in future studies.

Procedures that are of great importance to develop include microfluidic systems for separation, collection and detection for use in critical resources like marines, sediments, soil, and food products in order to minimize or prevent harm to natural resources. Plastic production is rising at an increasing rate, as a result of the growing human population and its associated needs. Microfluidics is an advanced technology, and is considered a next-generation strategy for disease monitoring and point-of-care applications [83]. Recent advances in microfluidics have focused on its capacity for in-field application in low-volume, easy-to-manufacture, and low-cost solutions that can be applied in any desired environment with a diverse variety of materials and sensors that can be easily integrated.

From the perspective of MP detection strategies, microfluidic platforms also have some limitations that still need to be addressed for the future in order to incorporate systems integrating microfluidics into MPs research. As a starting point, the production of microfluidic systems faces many challenges. Although well-established lithography, embodying, and printing systems are extensively utilized for their fabrication, an ideal production scheme would have a better resolution during production, would be cost effective, and would be able to be scaled up for the mass production of necessary components [84]. In the future, 3D printing will provide the necessary infrastructure for the batch production of microfluidic systems able to detect and characterize MPs [85]. From the perspective of the incorporated modules (e.g., sensors), microfluidic systems may also possess disadvantages in terms of sensitivity and specificity. Microfluidic systems can not only be used for the determination and isolation of MPs, but also for the separation of algae, pathogenic bacteria and fungi, proteins, and rare metals, which directly affect the analysis of MPs [86,87]. This could lead to false positive results, since their sizes and size ranges are similar to those of microplastic particles. On the other hand, the samples themselves could quench fluorescent dyes [88]. Moreover, other detection techniques, including FT-IR, may also require concentrated samples of microplastics, which could lead to the channels of the microfluidic systems becoming clogged [89]. Overcoming these difficulties will increase the efficiency of carrying out studies, as well as enabling stronger measures to be taken against the threat of microplastics.

In conclusion, MP pollution has great importance in terms of ecosystem and human health, and the dangers resulting from an increase in this pollution around the world should be identified as soon as possible. Microfluidic systems have significantly increased the efficiency of strategies for the detection of these impurities. The integration of microfluidic systems with other microscopic and spectroscopic methods has also increased confidence in this technology. These systems can be used not only for these impurities, but also for the determination of many targeted molecules at the micro and nano scale. The necessary steps for the development of these platforms should be taken in such a way that this technology can be made available to consumers. The fact that these studies describe platforms that are able to shed light on the future for such pollutants and the characterization and cleaning of pollution globally is a big step toward a green earth in the future.

## Figures and Tables

**Table 1:** Conventional detection and characterization methods for MPs.

Instruments	Advantages	Disadvantages	References
Optical Microscopy	Capable of being modified with fluorescent and affinity-based dyes. Rapid; easy-to-use and inexpensive equipment.	Size and shape information are limited. Tracking transparent particles is difficult. Biological samples may also show signals with dyes. Sediment samples or other contaminants may interfere with the results and reduce reproducibility	[53]
Scanning Electron Microscopy (SEM)	Low detection limit. Elemental analysis is possible with energy dispersive X-ray spectroscopy (EDS) and electron energy-loss spectroscopy (EELS).	The instrument is highly expensive, and requires specialized training. While elemental analysis can be performed, the type of polymer cannot be determined exactly.	[43]
Transmission Electron Microscopy (TEM)	Ultra-low detection and resolution limit are provided. Elemental analysis is possible with EELS. It is also suitable for nanoplastic studies.	The device is highly expensive, and trained personnel are required. Sample preparation processes are complicated. While elemental analysis can be performed, the type of polymer cannot be determined exactly.	[45,54]
Atomic Force Microscopy (AFM)	Sample surface analysis can be performed. The sample surface damage is minimal. Low detection limit is exhibited. Stiffness, hydrophobicity, conductivity, and magnetization analysis can be carried out in addition to size measurements.	Contact mode, non-contact mode, and tapping mode have their own difficulties depending on the material type. The mode selection is critical. Sediment samples or other contaminants may cause errors in the results.	[55,56]
Fourier Transform Infrared (FT-IR) Spectroscopy	Chemical composition of MPs can be analyzed. Sample integrity is conserved.	Through the analysis with the aid of KBr, material recovery is difficult. There may be interactions between KBr and the substances. Compounds such as moisture and carbon dioxide cause different peaks to be observed in the spectrum. Expensive infrastructure is required.	[57]
Raman Spectroscopy	Analysis for micro- and nano-sized plastics can be conducted. Detection can be applied to many surfaces and sample phases. Chemical analysis of the particles can be performed.	The process is time consuming. Expensive instrument required.	[58]
Pyrolysis Gas Chromatography-Mass Spectrometry (Pyro-GC/MS)	Polymer type and additional contaminants could be tested together.	Data type and characterization procedure are complex. Sample recovery is not possible. It is insufficient to obtain information about the size.	[35]
Nuclear Magnetic Resonance (NMR)	MPs with different size and shapes can be analyzed. Material-specific characterization is possible. Process is time efficient.	Procedures for sample preparation are complicated. Device infrastructure is costly. The sample may be destroyed by dissolving.	[59,60]

**Table 2:** Separation, isolation, and detection of MPs using microfluidic technologies.

Source of MPs	Properties of MPs	Aims of Process	Properties of Microfluidic Platform	Detection Strategies	Validation Parameters, System Advantages, and Highlights of Process	Ref.
Soil	Average diameter of 35 µm.	Capturing of PE pollutants using film thickness. Investigating the motions of MPs at different flow rates and particle volumes.	A PDMS-based chip was designed with a channel width of 400 µm.	An inverted optical microscope was integrated for the quantification and the display of MPs behaviors.	*P* value was calculated as 0 in the limit of critical number of capillaries (≤1.3 × 10^−4^). The increase in the film thickness improved the capture efficiency of MPs.	[74]
Soil	PS MPs with sizes of 1 µm at concentrations of 100 mg/L and 1000 mg/L.	Evaluating the effects of PS on egg-laying of *Caenorhabditis elegans* (*C. elegans*)	A PDMS-based chip was designed with 8 channels (width of 85 µm). The chip also integrated fluorescence imaging.	Fluorescence imaging was utilized for the detection and monitoring of *C. elegans* GFP expression and Nile Red-stained MPs.	The analytical limit of the designed device was 40 worms/h. Significant reduction in the egg-laying and size of the worms was observed with the implementation of a PS MP concentration of 1000 mg/L.	[76]
Soil	PS MPs with a size of 1 µm with different labels (red, green, and blue fluorescent).	Investigating the impact of PS MPs on thrombosis.	A PDMS-based chip was designed with channels with a width and height of 100 µm. The chip incorporated a fluorescence imaging platform.	A confocal microscope was integrated for fluorescently stained MPs, and the monitoring of MPs with respect to the thrombosis effect.	The consistency of the experiment was demonstrated by obtaining a *p*-value lower than 0.0001. MP-invaded thrombosis and normal thrombosis were compared using Bland–Altman analysis (n = 16), which revealed a mean bias of 69.10 mg/2 mL with a standard deviation (SD) of 17.43. MPs bound to fibrins reduced the binding between fibrins and platelets. Hence, the formation of thrombosis was demonstrated.	[79]
Wastewater	PS with a diameter of 15 µm, and PET and nylon 6 fibers with a length of 200 µm.	Collecting MPs in the middle of the channel.	A Pyrex glass microfluidic chip with channels having a width of 707 µm and a depth of 505 µm was designed. This platform incorporated an acoustic focusing device.	Nile Red-stained MPs were collected using the acoustic focusing strategy, and these collected pollutants were displayed and analyzed via fluorescence microscopy.	Bulk Acoustic Wave (BAW) provided an enhanced limit of detection (LOD) for the collection of MPs with a diameter of 1 µm. MPs with a diameter of about 5 µm were collected effectively.	[65]
Wastewater—laundry outlets	MPs with diameters varying between 6 and 300 µm.	Examining the effect of medium density and particle size on the collection of MPs	A PDMS-based microfluidic platform was designed with steel tube channels possessing a width of 484 µm.	MPs were collected using an acoustic focusing strategy, and epifluorescence microscopy was implemented for dye-free MP monitoring. An automated cell counter was used for MP concentration measurement.	After 10 serial separations were carried out, the results demonstrated 90% reliability. MPs smaller than 180 µm in diameter were isolated with a higher yield.	[66]
Seawater, deep-sea sediments, and food containers	MPs with an average diameter of 19 µm.	Collection of MPs at the outlet of the microfluidic system.	A PDMS-based chip was designed with channels with a width of 500 µm and a height of 220 µm. The chip was combined with optical photothermal infrared spectroscopy (O-PTIR).	MP monitoring and size measurement were carried out using fluorescence microscopy and particle recovery rates (PRRs), respectively. In addition to size determinations, O-PTIR and Raman spectroscopy were utilized for the chemical analysis of MPs.	This device exhibited 90% separation and detection efficiency for MPs larger than 19 µm in diameter. *p* value was calculated as <0.01 with the implementation of Welch’s *t*-test for all independent experiments. The recovery rate of MPs was higher than that of density separation and filtration methods.	[10]
Tea bags	MPs with a diameter of 10, 20, and 30 µm.	Detection of size and quantification of the concentrations of MPs.	A PDMS-based chip was designed with a channel with a width of 750 µm and a length of 400 µm. It incorporated resistive pulse sensors (RPSs).	Optical microscopy and scanning electron microscopy (SEM) were used for MP observation. Resistive pulse sensors (RPSs) were utilized for MP size measurements.	A 10-fold increase in sensitivity allowed the device to identify particles with a diameter of 2 µm and a lowest concertation of 14 particles/mL. The average size and concentration of the MPs were measured as 21.9 µm and 6.52 × 10^4^ particles/mL, respectively.	[73]
Water	PS MPs with various densities (2 × 10^8^, 1 × 10^9^, 3 × 10^9^, and 6 × 10^9^ g/mL).	Collection of PS MPs on the HMDS-coated wells and detection using terahertz spectroscopy.	A PDMS-based chip was designed with channels with a width of 2 mm and a height of 20 µm, including wells with a width of 3 µm and a depth of 10 µm.	Terahertz spectroscopy was used in determining the densities of captured PS via alterations in frequency.	For number of PS < 30, the sensitivity coefficient α was calculated as 7.1 × 10^−4^. In situ PS detection was accomplished by continuous monitoring via terahertz spectroscopy. This system provides a cost-efficient strategy.	[77]
Crumbled coffee and yogurt cups	MPs with a maximum detected size of 125 × 81 µm in the solution.	Imaging of MPs performed for structural analysis and measurement of the number of particles.	A PMMA-based microfluidic platform was fabricated with channels with a width of 1000 µm and a height of 200 µm. This platform incorporated a 3D hologram microscope.	3D hologram microscope for the monitoring of MPs, and the measurement of their size and morphological properties.	MPs were separated, and their dimensions and shapes were further characterized.	[75]
Cotton and Acrylic synthetic fibers, storage containers, and yeast	PE MPs ranging in size from 10 µm to 45 µm, and PS MPs ranging in size from 9.5 µm to 11.5 µm.	Increased selectivity and sensitivity for MPs was achieved using Nile Red fluorescent dye.	A 3D-printed PDMS microfluidic system was fabricated with channels with a length of 400 mm. The 3D-printed platform incorporated an inverted microscope.	Nile Red-stained MPs were monitored with an inverted microscope, and ImageJ software was utilized for MPs identification.	The use of-spherical MPs resulted in higher-quality fluorescence. The highest-intensity fluorescence was obtained at the lowest flow rate and the highest temperature.	[64]
Commercial Products; PS (Baseline Chromtech, Tianjin, China), PE (Alfa Aesar, USA), and PP (Kingao Chemical, Hubei, China)	PS with a diameter between 50–200 µm. PE with a diameter of 500 µm. PP with a diameter between 50 and 150 µm.	Detection of MPs using Nile Red fluorescent dye.	A PMMA-based microfluidic platform was designed. The channels were fabricated with a width of 400 µm and a height of 500 µm. FT-IR was integrated with this platform.	Nile Red-stained MPs were monitored under a fluorescence microscope, and chemical composition analysis was performed using FT-IR spectroscopy.	The limit-of-detection (LOD) was determined as a diameter of 20 µm for stained MPs. Video and continuous monitoring provided the advantage of real-time detection of particles. The number of MPs, size, and their mobility were measured efficiently.	[70]
Commercial Product; PMMA and PS (Thermo Fisher Scientific, Duke Scientific, Polysciences Inc. and Microbeads AS)	80 µm size of PS, and 20 µm size of PMMA	Separation and characterization of PS and PMMA MPs using Raman and FT-IR spectroscopy.	A PDMS-based microfluidic chip was combined with spectroscopic methods.	Monitoring and classification with image processing of MPs were performed with a camera and IDEAS Analysis Software, respectively. FT-IR and Raman spectroscopy were utilized for the chemical analysis of MPs.	The LOD ranged down to MPs with a diameter of 20 m. MPs were separated and collected using the size sorting method. In this region, analyses were performed using spectroscopy techniques to characterize the chemical structures of MPs.	[48]
Commercial Standards	PMMA with a size of 40 ± 18 µm, PS with a size of 39.5 ± 1 µm, LDPE with a size of 300 µm (irregular).	Anti-Stokes Raman Scattering and Two-Photon Excited Autofluorescence Analysis were used for detection and separation of MPs.	A quartz glass flow cell with an inner thickness of 500 μm, a width of 8 mm, and a length of 40 mm was integrated with Raman spectroscopy.	Coherent Anti-Stokes Raman Scattering (CARS) was utilized to detect MPs, and their amount was calculated through relevant fluorescence intensity analysis.	Analysis of water-submerged targets is possible.	[71]
Commercial Standard	PE with sizes of 212−250 μm, 300−355 μm, 425−500 μm, 500−600 μm, 600−710 μm, and 850−1000 μm.	Impedance measurement for the detection of microplastics.	Gold-plated circuit boards as electrode bases, supported by acrylic and epoxy, were utilized.	Impedance spectroscopy was used to understand the size and material characteristics of MPs by measuring and utilizing the electrical properties of pollutants.	This system provided high-throughput and real-time measurement.	[72]
Commercial Standard	Carboxyl-functionalized PS beads with a size of 1 mm.	Electrochemical separation of microplastics observed with optical imaging.	A glass/PDMS microfluidic chip with a length of 15.0 mm, a width of 100.0 mm width, and a microchannel with a thickness of 6.0 mm was fabricated using the photolithography method.	An inverted microscope was implemented for the real-time monitoring of MPs. COMSOL simulation was performed for the detection of MP behaviors during the separation process.	Continuous separation was achieved.	[67]

## Data Availability

Not applicable.
